# University of City of New York

**Published:** 1881-11

**Authors:** 


DR. FREDERIC R. STURGIS was recently elected clinical Professor of Venereal Diseases in the University of the City of New York. A good appointment.

